# A Possible Drug-Drug Interaction Between Eliquis and Amiodarone Resulting in Hemopericardium

**DOI:** 10.7759/cureus.13486

**Published:** 2021-02-22

**Authors:** Abdulbaril Olagunju, Mohammad Khatib, Frances Palermo-Alvarado

**Affiliations:** 1 Internal Medicine, Creighton University School of Medicine, Phoenix, USA

**Keywords:** hemorrhage, pericardium, effusion, eliquis, amiodarone, cytochrome p450, p-glycoprotein

## Abstract

According to the ARISTOTLE trial, apixaban was superior to warfarin because it was associated with fewer strokes, systemic embolism, and bleeding. Hemopericardium was one of the major bleeding events reported in this trial. However, the percentage of patients that developed hemopericardium was not stated in the trial results.

We present a case of hemopericardium in an 80-year-old man admitted for dyspnea, cough, and lower extremity edema. He was recently diagnosed with paroxysmal atrial fibrillation and started on apixaban for stroke prevention. Prior to admission, he was taking metoprolol succinate and amiodarone for atrial fibrillation. His symptoms resolved after undergoing successful pericardiocentesis.

Although hemopericardium is a rare side effect associated with the use of non-vitamin K oral anticoagulants (NOACs), we suspect that a drug-drug interaction between apixaban and amiodarone (via the cytochrome p450 system and p-glycoprotein efflux pumps), the patient's advanced age, and borderline creatinine are possible risk factors.

## Introduction

Apixaban is a non-vitamin K oral anticoagulants (NOACs) in the same class as rivaroxaban, endoxaban, and dabigatran. It is licensed in the United States for the prevention of stroke in nonvalvular atrial fibrillation and venous thromboembolism [1]. Although it was associated with a greater reduction in the rate of stroke and a lower rate of bleeding compared to warfarin in the ARISTOTLE trial, it was associated with an increased risk of major bleeding (including hemopericardium) defined using the International Society on Thrombosis and Hemostasis (ISTH) criteria; at an incident rate of 2.13% per year [2,3]. However, the percentage of hemopericardium compared to other bleeding sites was not included in the trial results [2,3]. Hemopericardium is the accumulation of blood in the pericardial space [4]. This could lead to life-threatening hemodynamic compromise, cardiac tamponade depending on the rate and volume of blood accumulation [4]. Reported causes are infection (especially tuberculosis), metastasis of malignant cells to the pericardium, thoracic aortic dissection, cardiac surgery, acute myocardial infarction, trauma, pericarditis, and bleeding diathesis [3]. We present a case of hemopericardium in a patient taking apixaban for paroxysmal atrial fibrillation.

## Case presentation

**Figure 1:**
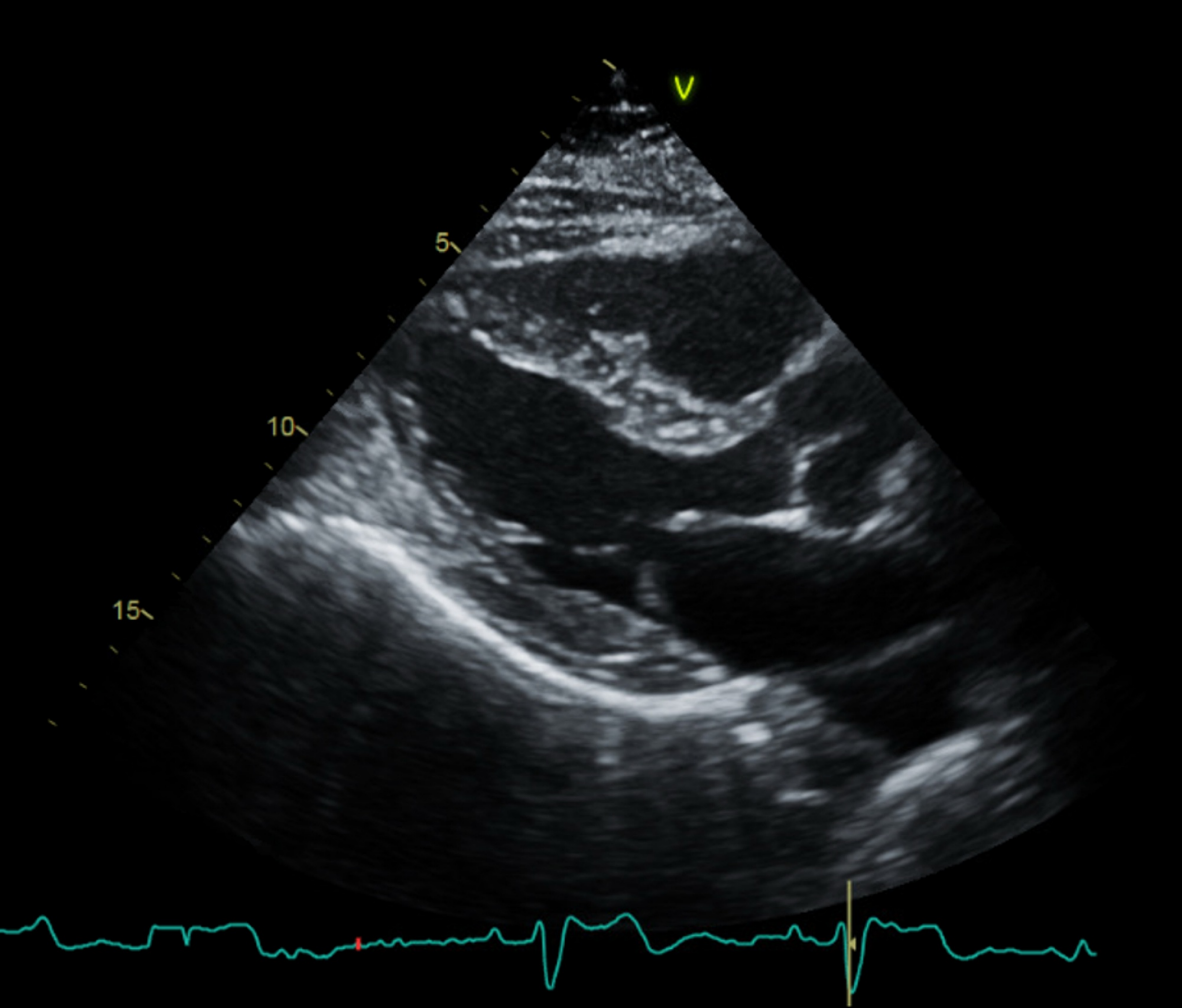
Parasternal long-axis view of the patient's baseline echocardiogram did not show pericardial effusion.

An 80-year-old male with a past medical history of paroxysmal atrial fibrillation, heart failure with preserved ejection fraction, chronic obstructive pulmonary disease, tobacco dependence, benign essential hypertension, and stage 3 chronic kidney disease presented to the emergency department (ED) with shortness of breath, orthopnea, increasing lower extremity edema, and cough of two weeks' duration. The cough was productive with white sputum and worse in the supine position. He denied hemoptysis, chest pain, fever, chills, night sweat, weight loss, nausea, and vomiting. His paroxysmal atrial fibrillation was diagnosed two weeks prior when he was admitted and treated for COPD exacerbation and cellulitis of the right shin. He converted to sinus rhythm after receiving 20mg IV diltiazem. Based on his CHA2DS2VASc score of 4, he was discharged home with 5mg twice a day (BID) of apixaban and metoprolol succinate 12.5mg BID for rate control; he was also started on amiodarone 200mg daily outpatient. A transthoracic echocardiogram before discharge revealed a normal ejection fraction of 62% with grade 2 diastolic dysfunction (Figure 1).

**Figure 2:**
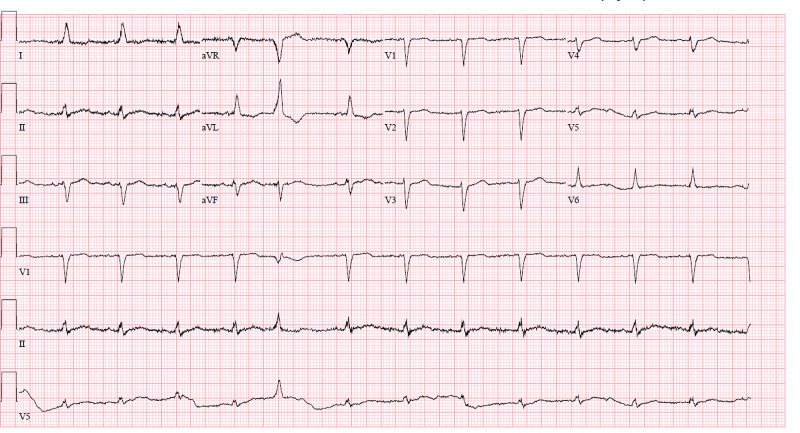
Electrocardiogram unremarkable for low QRS voltage or electrical alternans.

**Figure 3:**
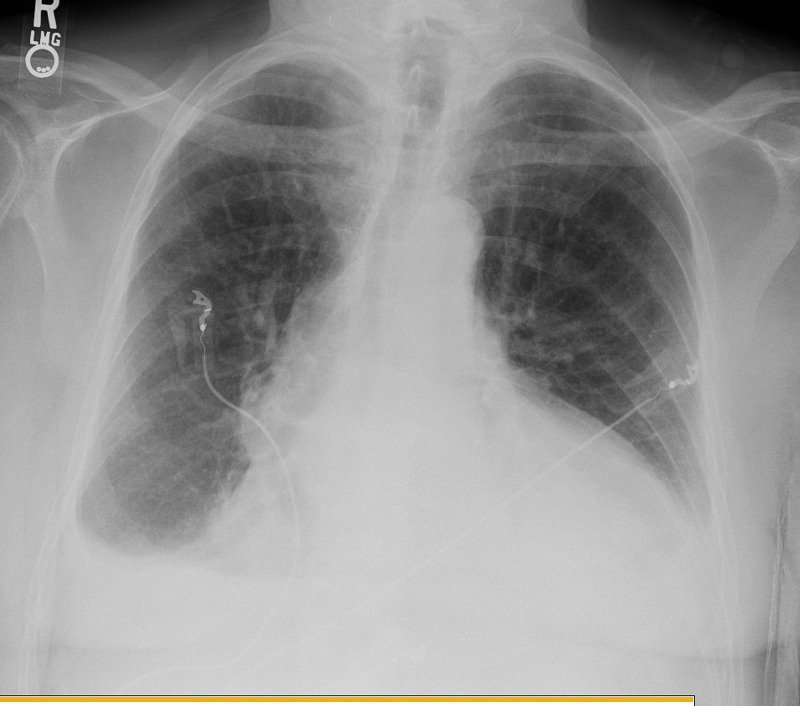
Chest radiograph remarkable for enlarged cardiac silhouette, new bibasilar airspace opacities, and small bilateral pleural effusion.

**Figure 4:**
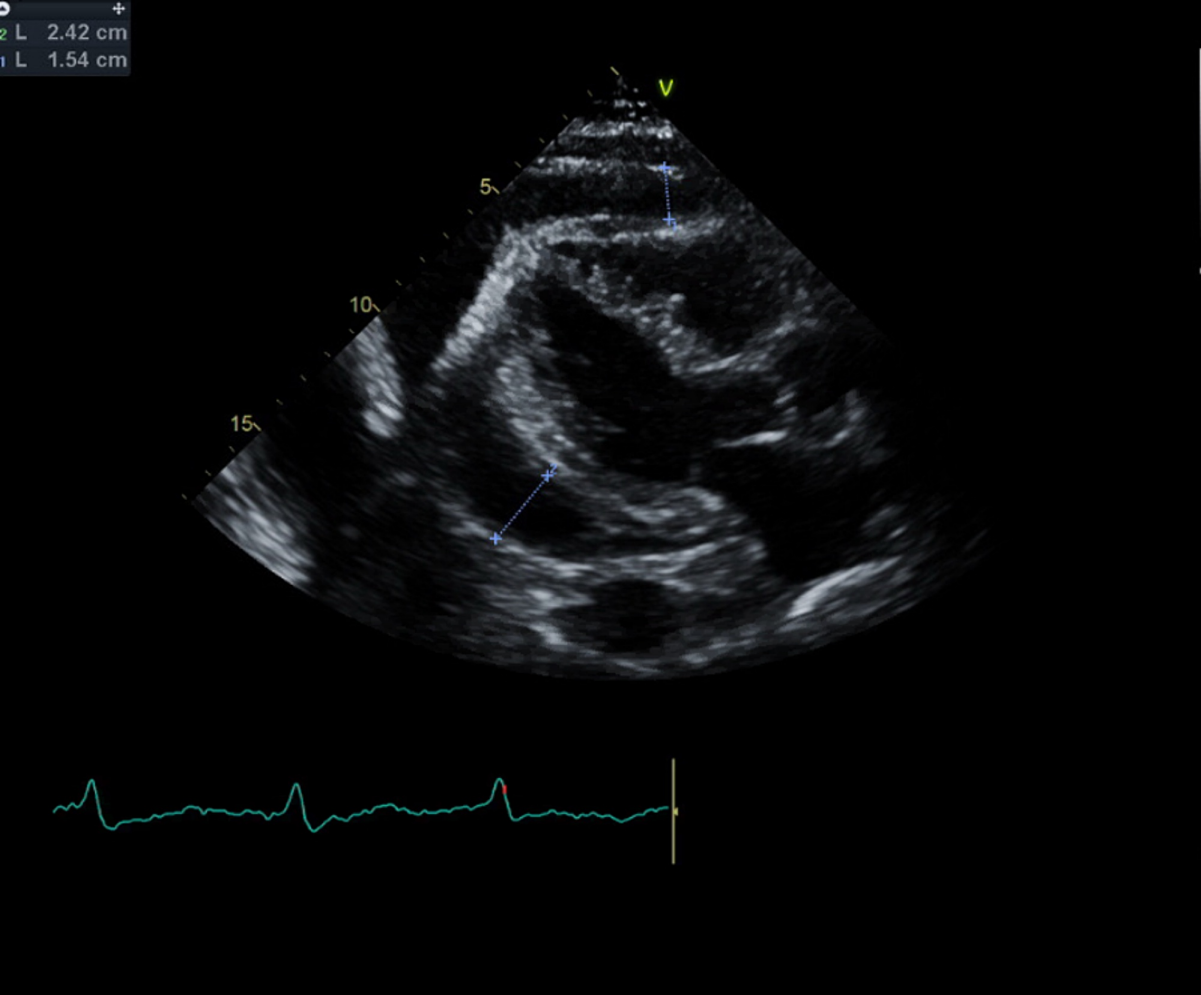
Parasternal long-axis view on echocardiogram remarkable for a large pericardial effusion.

On presentation to the ED, he was tachypneic with a respiratory rate of 32 breaths per minute and oxygen saturation of 91% on room air, his heart rate was 77 beats per minute and blood pressure was 114/78mmHg. Physical examination was remarkable for jugular venous distention, distant heart sounds, and 3+ bilateral lower extremity edema up to his shins. Abnormal laboratory findings on admission were hemoglobin of 10.8g/dL (his baseline is 12g/dL), creatinine of 1.67mg/dL (his baseline is 1.42mg/dL), glomerular filtration rate (GFR) of 40mL/min (his baseline is 50mL/min), blood urea nitrogen (BUN) of 31mg/dL, INR of 3.0, and N-terminal brain natriuretic peptide (NT-BNP) of 729pg/mL. Electrocardiogram revealed sinus rhythm with premature ventricular contraction, no low QRS voltage or electrical alternans (Figure 2). The chest radiograph was remarkable for an enlarged cardiac silhouette, new bibasilar airspace opacities, and small bilateral pleural effusion (Figure 3). A limited bedside echocardiogram was remarkable for a large pericardial effusion with fibrinous material noted in the effusion, an excessive respiratory variation of the mitral and tricuspid inflow velocities, a dilated inferior vena cava (IVC) with a diameter >2.1cm, and <50% respiratory collapse estimating a right atrial pressure of 15mmHg. There was no collapse of the right ventricle (Figure 4).

He immediately received 50 units/kg of prothrombin complex concentrate to reverse the effect of apixaban and urgent pericardiocentesis was performed. There was 600mL of frank blood initially drained and an additional 200mL was drained by the end of the following day via the pericardial drain. The pericardial fluid was sent for cytology, acid fast bacilli (AFB) culture, stain, fungal culture, gram stain, and bacterial culture. A repeat echocardiogram a day after pericardiocentesis revealed complete evacuation of the effusion. Although the pericardiocentesis led to an improvement in his shortness of breath and orthopnea, he required diuresis with 20mg of furosemide for complete resolution. The pericardial drain was removed on day 3 of admission and he was discharged the following day.

Cytology of the pericardial fluid revealed numerous red blood cells, lymphocytes, neutrophils, and benign mesothelial cells. There were no malignant cells. There were no organisms on gram stain and bacterial culture was negative after five days. No organisms were noted on the AFB stain; AFB culture remained negative after five weeks. Fungal culture remained negative after four weeks. 

## Discussion

The absence of malignant cells in the pericardial effusion, negative cultures, and stains makes apixaban a possible cause of hemopericardium in this patient. Drug-drug interaction with amiodarone, the patient's advanced age, and borderline creatinine might explain this finding. 

Apixaban has a half-life of 12 hours [5]; approximately 25% is eliminated indirectly into bile as hydroxylated-O-demethyl apixaban after metabolism by the cytochrome p450 system, particularly CYP3A4 [6]. About 25%-27% is eliminated directly by the kidney and the rest is secreted directly into bile and excreted into feces via the p-glycoprotein efflux pump [6,7]. Amiodarone is a known inhibitor of CYP3A4 and the p-glycoprotein efflux pump [7-11]. It likely increased the half-life of apixaban in this patient and possibly contributed to the hemopericardium. Interestingly, about 11.4% of the patients in the ARISTOTLE trial received amiodarone [12]. In addition, according to a post-hoc analysis of the ARISTOTLE trial, a retrospective study of p-glycoprotein inhibitors and apixaban, the bleeding outcomes were not statistically significant when compared to patients taking apixaban alone [10].

The patient's advanced age and stage 3 chronic kidney disease could have also predisposed to hemopericardium. Perhaps a dose reduction to 2.5mg BID upon initiation of amiodarone might have prevented this. However, there are no guideline recommendations that back this up. Based on the apixaban dosing guidelines, the 2.5mg BID dosing should be used in patients with two of three criteria which are age, weight, and creatinine of at least 80 years, 60kg, and 1.5mg/dL, respectively [13]. Our patient’s weight of 72.6kg and baseline creatinine of 1.42mL/min at the time of apixaban initiation meant that the patient met only one of the three criteria. Perhaps the impact of age on the likelihood of bleeding is independent of the presence of the other two criteria.

## Conclusions

This is a very unusual case of hemopericardium because both amiodarone and apixaban are commonly prescribed together. A drug-drug interaction between apixaban and amiodarone could have increased the risk of bleeding in this patient via inhibition of the cytochrome p450 system and p-glycoprotein efflux pumps. In addition, the patient's age of 80 years and borderline creatinine of 1.42mg/dL might have also predisposed him to bleeding.
